# Preventing Pesticide Toxicity Risk Through Self-Reported Practices in Children of Farming Communities: A Social Practice Theory Perspective

**DOI:** 10.3390/jox16030117

**Published:** 2026-06-22

**Authors:** Nuraeni Nuraeni, Herdis Herdiansyah, Fatmah Fatmah, Haruki Agustina, Rully Yusuf

**Affiliations:** 1 Department of Environmental Science, Graduate School of Sustainable Development, Universitas Indonesia, Central Jakarta 10430, Indonesia; nuraeni42@ui.ac.id (N.N.); ffatmah@yahoo.com (F.F.); haruki.agustina11@ui.ac.id (H.A.); 2 PT Pegadaian (Persero), Central Jakarta 10430, Indonesia; yusuf.rully168@gmail.com

**Keywords:** child, pesticide, social practice theory, risk, farming family

## Abstract

This study analyzes the determinants of self-reported behaviours and perceptions associated with pesticide toxicity risk in children using the Social Practice Theory framework, linking individual factors and agricultural practices to understand vulnerability and prevention opportunities. This research was conducted in Pattapang Village, Tinggimoncong District, Gowa Regency, South Sulawesi Province, Indonesia. To examine the relationship between pesticide use patterns, social norms, competence, material, and individual aspects and the risk of sensitive toxicity in children, data were analyzed using structural equation modeling-partial least squares (SEM-PLS) with bootstrapping resampling. Pesticide use patterns had a significant negative effect on toxicity risk. Competence was the strongest predictor of pesticide use patterns, followed by materials and short-term goals. Personal values dominate personal norms and long-term goals, while social norms only influence personal norms. Self-efficacy, personal norms, and long-term goals showed no significant effects. The novelty of this research lies in the integration of a socio-ecological approach with individual psychological factors in a comprehensive structural model that explains the complex mechanisms of children’s protective behavior formation from pesticide toxicity, identifying that personal values—not personal norms or self-efficacy—are the most effective leverage points for farmer behavior change interventions.

## 1. Introduction

Pesticides with high toxicity, persistence in the environment, and a tendency to bioaccumulate pose significant health risks, particularly to vulnerable groups such as children [1,2]. Toddlers have unique physiological and behavioral characteristics that make them more vulnerable than adults to pesticide exposure. Exposure to hazardous chemicals can compromise children’s developing immune system, nervous system, and vital organs [3]. Deficiencies in detoxification enzymes—particularly paraoxonase and chlorpyrifos-oxidase—compared with the adult population [4] result in significant vulnerability to the pesticide-related health effects in children. This can be seen from epidemiological data from South Africa, which shows that 42.6% of deaths from pesticide exposure occur in children under the age of five [5]. Meanwhile, in Indonesia, Ibrahim & Sillehu [6] recorded a prevalence of pesticide exposure of 35.4%. The habit of playing in the dirt, putting objects in their mouths, and a lack of parental awareness of environmental hazards increase the risk of pesticide exposure in children [7].

Pesticide poisoning can be acute—with symptoms such as dizziness and skin irritation [8,9,10]–or chronic, which can lead to leukemia and brain tumors [7]. The impact of pesticide exposure can be linked to the incidence of cancer in children [11,12,13,14]. According to a report by Marquez [15], the incidence of childhood cancer increased by 36% in 2012 compared to 1975.

It is widely recognized that children should not have direct contact with pesticides; however, indirect exposure remains an unavoidable reality in agricultural communities due to their proximity to agricultural activities and the behavior of adult caregivers in shared living spaces. This condition indicates the limitations of research on pesticides and children. Based on this gap, this study conceptualizes three elements of social practice: meanings, competencies, and materials, along with individual factors [16]. This study addresses the issue of pesticide exposure in children in agricultural settings, which remains a significant concern in both developing and developed countries.

Protective behavior to reduce the perceived risk of pesticide exposure in children can be influenced by various social and psychological factors, as explained by Social Practice Theory and individual factors, as in research Kaiser et al. [17], although this research is still limited to exploring the routines of farmers as users. Social Practice Theory alone is insufficient to explain individual-level psychological factors such as self-efficacy and personal values, which are known to shape protective behavior; conversely, individual-level theory does not capture the collective and routine-based nature of agricultural practices. Therefore, integrating both frameworks addresses the gap between structural practices and individual agency in explaining self-reported pesticide-related protective behavior in farming communities.

The study focuses on household practices and exposure-related behaviors that contribute to the self-reported perceived risk of pesticide exposure in children, rather than measuring actual toxicity outcomes. Specifically, the study asks: “What are the influences of pesticide use patterns, meanings, competencies, materials, and individual factors on self-reported exposure-related behaviors that contribute to the potential risk of pesticide exposure in children in agricultural settings?” Therefore, this study aims to analyze the influence of these factors on self-reported exposure-related risk-related behaviors. These findings are expected to contribute to the international literature on environmental health, child vulnerability, and protective behavioral practices in agricultural contexts.

### 1.1. Individual Factors

At the individual level, factors such as personal norms [18], personal values, long-term goals, and self-efficacy play a significant role in determining individual decisions [17]. However, some farmers may be aware of the dangers of pesticides to their families’ health but feel they lack the choice or capacity to change their farming practices due to economic pressures or concerns about reduced crop yields [19]. This can occur when farmers have low self-efficacy. Self-efficacy refers to a person’s belief in their ability to perform certain behaviors to achieve desired outcomes [20]. In the context of protecting children from pesticide exposure, farmers’ self-efficacy relates to their belief that they are capable of implementing safer pesticide use practices and protecting their children from the risk of exposure. However, if farmers feel that they lack the ability or resources to implement safer practices, these personal norms are less likely to translate into action. In other words, even if they know what to do, low self-efficacy can prevent them from acting on those values. Conversely, farmers with high self-efficacy—who feel they can handle pesticides safely, use personal protective equipment, or implement pesticide-free zones around their homes, are more likely to apply their personal values in their daily actions [21].

According to Schwartz, personal values can be categorized into two categories: self-transcendence values and self-enhancement [17]. In the agricultural context, farmers’ personal values regarding family welfare and concern for future generations can influence decisions regarding pesticide use. Values do not directly drive behavior but rather form the foundation of personal norms—a sense of moral obligation to act in a certain way. Farmers who value children’s health will develop a strong personal norm to limit the use of hazardous pesticides. Farmers with intergenerational responsibility values will also develop a goal of ensuring a safe agricultural environment for their children.

Individual goals in the context of preventing pesticide exposure in children can be divided into immediate and long-term goals. Short-term goals refer to specific outcomes and processes related to agricultural practices that are relatively important to farmers in the short term [17]. An example is the desire to ensure that children’s play areas, such as gardens and yards, are free from pesticide sprays. Meanwhile, long-term goals refer to broader aspirations and objectives that farmers hope to achieve over a longer period of time. These long-term goals are often linked to an individual’s value system and may include the desire to pass on a viable agricultural legacy to the next generation, including good soil conditions that can be achieved by reducing the use of agrochemicals [17]. Those who are more oriented toward short-term goals, for example, high crop yields to meet daily needs, are more likely to maintain pesticide use despite awareness of the risks, including those for children.

Personal norms can influence farmers’ decisions to limit pesticide use based on moral considerations such as responsibility for others’ health and safety or concern for environmental sustainability [17]. Personal norms can also be strengthened by experiencing events that can pressure or perpetuate individual decisions. For example, Yang et al. [22] found a significant shift in their perspectives on agricultural practices when farmers began to realize the consequences of toxic pesticide use on the sustainability of agroecosystems. This awareness emerged with a deeper understanding of the risks of excessive pesticide use—which not only damages soil quality and endangers consumer health. This view evolved into a personal commitment to switch to more environmentally friendly pest control methods over time. This transformation suggests that individual motivation, driven by long-term interests and ecological values, can drive behavioral changes that may be more sustainable than interventions relying solely on external pressures.

### 1.2. Social Practice Theory: Meanings (Social Norms), Competence, and Material

Social Practice Theory is a theoretical framework for understanding human behavior that differs from conventional linear approaches [17]. Social Practice Theory can be defined a framework that explains routine behavior that incorporates a set of elements such as knowledge, skills, ideas, and meanings [16]. This approach shifts the focus from individuals or institutions to “bundles of practices,” or sets of interconnected elements, and examines how these practices are reproduced, sustained, stabilized, challenged, and transformed in social contexts [23]. Unlike individual-centered theories such as the Theory of Planned Behavior or the Theory of Reasoned Action, which rely on assumptions of rationality and linear decision-making, the Theory of Planned Behavior recognizes the complexity and dynamics of action [17].

The conceptual debate surrounding the Theory of Planned Behavior reflects the theory’s diverse schools of thought. Bourdieu emphasizes practice as the result of the interaction between habitus (dispositions), capital (resources), and field (social arena) [24]. Giddens developed structuration theory, which views social practices as routines reproduced by agents through rules and resources [25]. Schatzki also conceptualizes practice as a “bundle of activities” organized by practical understanding, rules, structures, and shared meanings [23]. Meanwhile, Shove proposes a simpler framework with three fundamental elements: materials, competences, and meaning [16]. This study adopts Shove’s conceptualization due to its balance between conceptual simplicity and strong analytical power, as well as its ability to integrate material and non-material elements in agricultural social practices.

Social practice theory has three main interacting elements: meaning, competence, and materiality [16]. Pesticide use is not only seen as an agricultural tool but also represents compliance with social expectations: being a “serious,” “productive,” and “successful” farmer [17]. This social perception makes pesticides part of farming identity and obscures the potential dangers that can affect families, including children.

In terms of competence, many farmers feel that they have a sufficient understanding of pesticide use. However, this understanding is often limited to the technical aspects of the application and does not encompass health impacts or environmentally friendly alternatives. This partial knowledge actually becomes a barrier to the adoption of new knowledge, especially if the information contradicts years of farming experience [26]. In this case, competence is not only about new knowledge but also about how that knowledge is received and interpreted in the context of the daily lives of farmers.

Families with lower economic status also tend to live closer to agricultural fields, increasing the risk of direct exposure for their children [27]. Furthermore, financial constraints may force parents to take their children to the fields, further increasing the exposure risk. Those who are more oriented toward short-term goals—for example, high crop yields to meet daily needs—are more likely to maintain pesticide use, despite awareness of the risks [28], including children. However, Bourguet and Guillemaud [14] showed that while the use of pesticides can increase agricultural productivity in the short term, the long-term health and environmental impacts are often overlooked. A comprehensive cost–benefit analysis that considers not only the short-term economic benefits of pesticide use but also the long-term health costs can provide a strong justification for investing in mitigation strategies. In this study, crop yields, pesticide expenditures, and child medical costs are conceptualized together under the “material” dimension of Social Practice Theory, as they collectively represent the economic resources and consequences that shape and constrain household pesticide-related practices. Although these variables differ in nature, they are united by their role as material conditions influencing pesticide use decisions and child protection behaviors.

### 1.3. Behavioral Patterns in Pesticide Use

The continued increase in pesticide use—despite the well-known negative impacts on human health and the environment—indicates that this issue is not merely a technical but also a more complex social issue. Pesticide use patterns, such as farmers’ reluctance to reduce or stop pesticide use, are closely related to farming styles that have become part of daily routines and socially established practices [29]. Conventional pesticide-based farming styles have been internalized as “normal” and even “professional” practices [30], and changes to more sustainable agricultural systems are often perceived as risks rather than solutions. Furthermore, these conditions increase the risk of pesticide exposure in children. However, this study assumes that wise and responsible pesticide use patterns—influenced by appropriate individual and structural factors—can reduce the self-reported perceived risk of pesticide exposure in children.

## 2. Materials and Methods

### 2.1. Research Location

**Figure 1:**
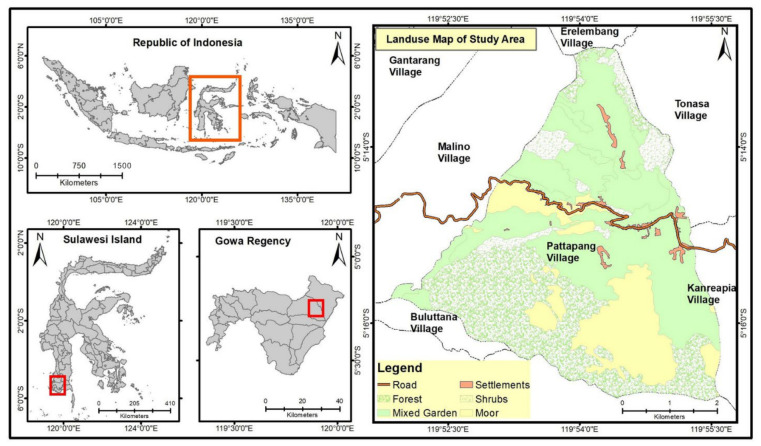
Land use map at the research location.

This research was conducted in Pattapang Village (Figure 1), an area with diverse topography ranging from lowlands to hills. Pattapang Village is a sub-district in Tinggimoncong District, Gowa Regency, South Sulawesi, Indonesia. It comprises four sub-villages, namely Pattapang, Kampung Beru, Lembanna, and Buluballea, covering an area of 142.87 km^2^ [31]. Topographically, Pattapang Village is located on the Malino Plateau at an elevation of 1300–1600 m above sea level, with slopes ranging from 15% to over 40%, and the highest point is near Mount Bawakaraeng. These conditions support the production of various food and horticultural crops. This location was chosen because it is the center of potato production in South Sulawesi [32] contributing 411,297 quintals or approximately 69.04% of total potato production in 2020 [32]. However, similar to many agricultural areas in Indonesia, Tinggimoncong District faces significant challenges related to excessive pesticide use—not following the recommended usage on packaging labels—and uncontrolled use [33].

In this region, most farming families live in wooden stilt houses, which can increase the exposure of children to pesticides. Wooden houses, which are often more porous than other types of houses, can allow greater infiltration of pesticide residues from nearby agricultural activities [34]. This condition is further exacerbated by the proximity of the house to fields or farmhouses where pesticides are actively used [35,36,37,38]. Preliminary findings indicate that farming families frequently visit or stay at farmhouses during the planting and harvesting seasons, with visits becoming more frequent closer to harvest. Children often accompany their parents to farmhouses. These conditions potentially increase the exposure of children to pesticide residues in and around these structures.

### 2.2. Population and Sample

This study limited the study population to only families with children under five, which numbered approximately 391 families based on 2025 data from the Tinggimoncong District Population and Child Protection Survey for 2025. The sample size calculation used the Krejcie-Morgan method with a 95% confidence level, indicating a minimum sample size of 195 respondents. A 10% increase was then made to prevent potential sample inadequacy due to respondents who could not be interviewed. Thus, the minimum sample size was 214 respondents, which was then rounded up to 215. Sample selection was performed using simple random sampling. The respondents in this study were either the primary caregivers of children under five years of age, mothers, or heads of households. However, the study data showed that fathers were the dominant respondents for several strategic reasons. Although mothers are generally the primary caregivers for toddlers, in the local cultural context, fathers are the dominant decision-makers in the family, including decisions regarding agricultural practices and pesticide use. Decisions regarding the type, frequency, and intensity of pesticide application in potato fields are generally determined by the head of the household. Furthermore, fathers have more detailed knowledge regarding spraying practices, application times, and the distance of the fields from the home—information that is crucial in assessing potential exposure among toddlers. Another consideration is that fathers, as direct actors in agricultural activities, have a better understanding of field conditions, including patterns of bringing toddlers to the fields, pesticide storage at home, and post-application hygiene practices, which can influence indirect exposure among toddlers. Therefore, data obtained from fathers is expected to more accurately describe the potential toxicity risks among toddlers in the context of potato farming families.

### 2.3. Data Collection

The potential risks of pesticide toxicity in children, pesticide use patterns, individual level, individual values, long-term goals, short-term goals, self-efficacy, social norms, competencies, and material needs were collected using a questionnaire (Table 1). This research questionnaire was modified from a questionnaire that had undergone validity and reliability testing in Deng et al. [39] and Kaiser et al. [17]. However, validity and reliability tests were conducted on the previously designed questionnaire. The following are the variables and indicators for this study:

### 2.4. Structural Equation Modeling—Partial Least Squares (SEM-PLS)

Data analysis was conducted using the Structural Equation Modeling with Partial Least Squares (SEM-PLS) method. SEM-PLS was used in this study because the research model is complex, with many latent variables and indicators having branching relationships (multiple pathways), and aims to predict the influence between variables. This method allows simultaneous analysis of direct and indirect influences within a single integrated model. Descriptive analysis and assumption testing were conducted using SPSS version 26.

**Figure 2:**
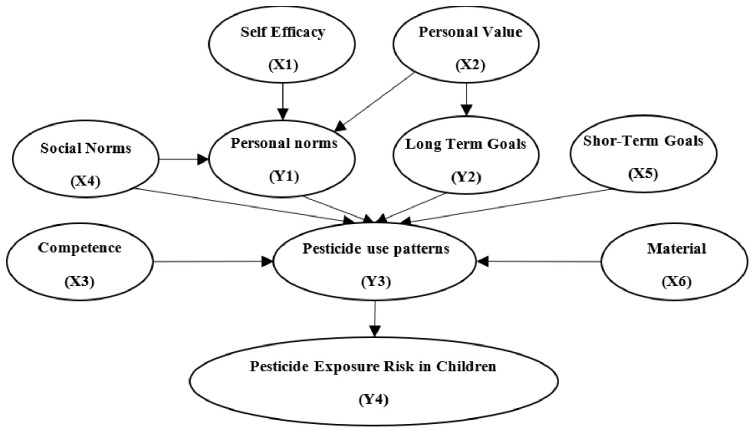
Research structure model diagram.

The SEM-PLS analysis stage begins with the definition of the measurement model (the influences between latent variables and their indicators) and the structural model (the influences between latent variables). The next stage includes model estimation, model evaluation (loading factor > 0.6; Composite Reliability > 0.7; Average Variance Extracted > 0.5; Fornell-Larcker criterion; cross-loadings), structural model evaluation, and hypothesis testing using the bootstrapping method [40]. Figure 2 shows the visualization of the structural model diagram of the research. The analysis was conducted using SmartPLS 4 (version 4.1.1.4). The following is the hypothesis of this research:

**H1:**  *Self-efficacy (X1) influences personal norms (Y1).*

**H2a:**  *Personal values (X2) influence personal norms (Y1).*

**H2b:**  *Personal values (X2) influence long-term goals (Y2).*

**H3:**  *Long-term goals (Y2) influence pesticide use patterns (Y3).*

**H4:**  *Short-term goals (X5) influence pesticide use patterns (Y3).*

**H5:**  *Personal norms (Y1) influence pesticide use patterns (Y3).*

**H6a:**  *Social norms (X4) influence pesticide use patterns (Y3).*

**H6b:**  *Social norms (X4) influence personal norms (Y1).*

**H7:**  *Competence (X3) influences pesticide use patterns (Y3).*

**H8:**  *Material (X6) influence pesticide use patterns (Y3).*

**H9:**  *Pesticide use patterns (Y3) influence the Pesticide Exposure Risk in Children (Y4).*

## 3. Results

### 3.1. Characteristics of Study Participants

Based on the characteristics of the respondents involved in this study, most respondents (90.2%) have one child, and their agricultural work experience varies considerably, with 55.3% having worked for more than 10 years. Respondents’ education levels showed that 42.3% had an elementary school education, 20.9% had a junior high school education, and 17.7% had a senior high school education, while only 2.8% had a college degree. The majority of respondents (95.8%) worked as full-time farmers. Pesticide sellers were the primary source of information for 45.6% of respondents, followed by fellow farmers (38.6%), family members (26.0%), and agricultural extension workers (24.2%). Very few farmers (1.4%) obtained information from social media. The age distribution of respondents shows a concentration in the productive age group, with 62.8% aged 25–29 years, followed by those aged 30–34 years (9.3%) and those aged 20–24 years (8.4%). This young age group can embrace innovation and changes in safer agricultural practices, but appropriate communication and education approaches are required (Table 2).

The geographic aspect, reflected in the distance of residence from agricultural areas, indicates a significant level of risk of exposure. The data revealed that 90.2% of respondents live within a 0–100 m radius of agricultural land, with only a small proportion living more than 500 m away. This geographic proximity creates conditions for direct and continuous exposure to pesticides not only for farmers but also for entire families, especially children. Homes adjacent to agricultural land can serve as entry points for pesticide contamination through air, water, and soil, exposing children even if they are not directly involved in agricultural activities.

**Figure 3:**
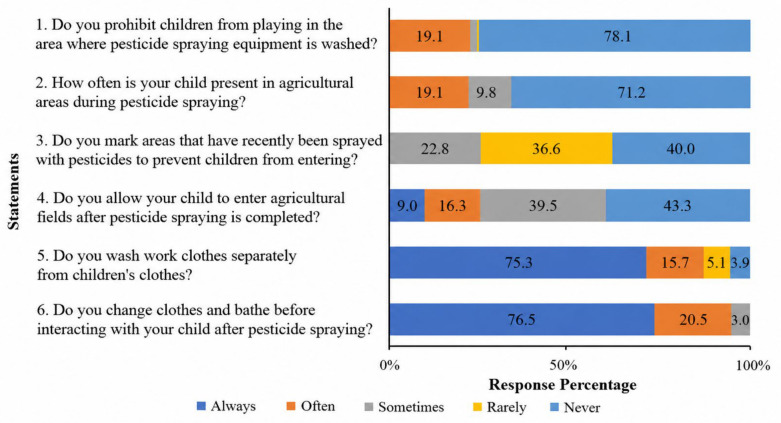
Pesticide Exposure Risk in Children.

The table above also shows that the majority of toddlers (83%) were most often at home when pesticides were first sprayed. In contrast, only 5% of toddlers were in the garden, and 12% were in the garden house. This study also highlighted variations in pesticide storage locations. Although 42% of respondents stored pesticides in the garden house and 76% kept them in the shed, 9% of respondents stored them outside or in a corner of the house, and 3% kept them inside the house. The self-reported pesticide exposure risk in children is illustrated by the fact that the majority of respondents (71.2%) did not allow toddlers to be in the agricultural area while pesticides were being sprayed. However, 38.8% of respondents still allowed their toddlers to enter the fields after spraying. The majority of respondents (78.1%) also never prohibited toddlers from playing in the sprayer washing area, and only 19.1% consistently enforced this prohibition. Meanwhile, 76.3% admitted to always changing clothes and bathing after spraying, but only 1% always marked the hazardous area after spraying pesticides (Figure 3).

From a health perspective, the data show that only 1.4% of respondents reported that their toddlers had experienced an acute respiratory infection (ARI), while 98.6% did not. However, higher rates were observed for other symptoms such as vomiting (24.7%), coughing (58.1%), fever (62.8%), and nausea (8.4%). It should be emphasized that these data reflect the history of symptoms experienced by toddlers, not the diagnosis of diseases directly attributable to pesticide exposure. Nevertheless, this health history information is important to document and analyze because symptoms such as fever, coughing, vomiting, and nausea are clinical manifestations that can arise from various causes, including pesticides. In addition to these common symptoms, several more serious health conditions were also reported, although at a lower prevalence. Dermatitis or skin problems were reported in 6.5% of toddlers, while asthma was recorded in 2.3% of respondents’ toddlers (Table 3).

### 3.2. Determinants of Pesticide Exposure Risk in Children

This study analyzed the self-reported pesticide exposure risk in children based on the influence of pesticide use patterns, meaning (social norms), competence, materials, and individual aspects. SEM-PLS modeling was used to demonstrate the complex relationships between variables [41]. This approach has been used in several studies on farmer behavior related to pesticide use [42]. An initial model was formed to obtain the Outer Loading value between the variables and their respective indicators. The results of the analysis showed that several indicators had Outer Loading values below 0.4, which needed to be eliminated according to the guidelines of Hair et al. [40].

Table 3 shows that several variables have AVE (Average Variance Extracted) values below 0.5, indicating the need for model improvement by eliminating indicators with low Outer Loadings. The analysis process then continued with several iterations, removing indicators with Convergent Validity (Outer Loading) values > 0.4. The indicators removed from the initial model to the final model are X12, C11–C16, C24–C26, SN11–SN13, SN21, SN23–SN24, XS4, M2, Y12–Y14, Y21, Y24, PPPF22, PPF31–PPF33, and PRT21–PRT25, based on the results of the validity and reliability tests during the indicator trimming process.

After going through the iteration process and eliminating indicators that did not meet the validity criteria, a final structural model with adequate validity and reliability test values was obtained. The results of the final model show improvements in terms of validity and reliability. All variables have Composite Reliability values > 0.7, indicating good construct reliability [40]. The AVE value for all constructs was also >0.5, indicating that all latent variable constructs in the final model were valid and reliable. Based on the Outer Loading results of the final model, all latent variable indicators in the final model were convergently valid because all Outer Loadings were >0.5 (Table 4).

After the measurement model (outer model) was confirmed valid and reliable, an analysis of the structural model (inner model) was conducted to test the causal relationships between latent variables. The following are the results of the coefficient of determination values for the final model (Table 5).

Based on the R-Square value (coefficient of determination) in Table 6, it can be concluded that (1) 17.2% of the variable Pesticide Use Pattern (Y3) is explained by variables in the model, with the remaining 82.8% influenced by external factors; (2) 47.3% of the variable Personal Norms (Y1) is determined by exogenous variables in the model (X1, X2, X4), and the remaining 52.7% is influenced by other factors outside the model. This R^2^ value is moderate and indicates that the combination of self-efficacy, personal values, and social norms has a substantial influence on the formation of farmers’ personal norms; (3) the variable Long-Term Goals (Y2) of 21.4% is determined by Personal Values (X2), and the remaining 78.2% is influenced by other variables. This relatively low R^2^ value indicates that the long-term goals of farmers can be influenced by other factors, such as economic, social, and structural factors, which are not included in the model. (4) The Pesticide Exposure Risk variable (Y4) is only 2.9% explained by Pesticide Use Patterns (Y3), indicating that pesticide exposure risk is more influenced by other factors that are not included in this study.

The square root of the AVE (bolded diagonal numbers) for each latent variable is greater than the correlation value of that latent variable with the other latent variables. For example, the square root of the AVE for variable X1 (Self-Efficacy) is 0.778, which is greater than its correlation with X2 (0.609), X3 (0.116), X4 (0.477), and the other latent variables (Table S1). The indicator loading factor values for their own latent variables (bolded numbers) are always greater than the correlation values for those indicators with other latent variables (Table S2). This study then conducted an assumption test as a requirement for Partial Least Squares analysis on the outer model to ensure the absence of multicollinearity. Table S3 shows that all indicators had VIF values < 5.

**Figure 4:**
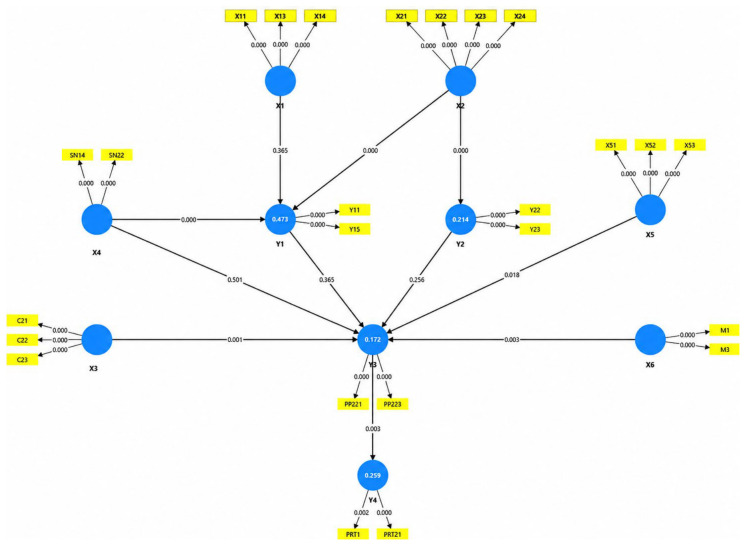
Model of relationships between variables through bootstrapping related to pesticide exposure risk in children. The arrows indicate the hypothesized directional relationships between constructs in the structural model.

This study also validated the fit of the constructed model or its suitability through a Goodness of Fit evaluation using several indicators. A model is considered fit if the Standardized Root Mean Square Residual (SRMR) value is <0.10 or 0.08, and the Normed Fit Index (NFI) produces a value between 0 and 1, with values closer to 1 indicating a better/fitter model (Hair et al.). [40]. Table S4 shows that the SRMR value for the saturated model is 0.086 (less than 0.10) and that for the estimated model is 0.098 (still below the 0.10 threshold). The NFI value is between 0 and 1 and is 0.486 for the saturated model and 0.255 for the estimated model, which is between 0 and 1. The combination of the NFI and SRMR values indicates that the constructed model is acceptable and meets the Goodness of Fit evaluation. The significant relationships between latent variables were tested using the Resampling Bootstrapping method with a subsample size of 5000, with significance defined as *p* < 0.05 [41]. The results of the path coefficient test on the final model are presented in Table 6 and Figure 4.

## 4. Discussion

### 4.1. Personal Norms and Intention-Behavior Gap

This study found that personal norms had no significant effect on pesticide use patterns (β = 0.090; *p* = 0.365), consistent with Kaiser et al. [17]. However, this finding contradicts Eskandar & Fatem [43], who found personal norms to be the strongest predictor of pro-environmental behavior, including pesticide use (*p* = 0.0001). Personal norms in the context of this study include guilt about using pesticides around children, discomfort with spraying pesticides near children, a sense of responsibility to prevent children from being exposed to pesticides, and regret and guilt if children become unhealthy due to pesticide exposure. This study found that the majority (41.4%) of respondents still did not feel responsible for preventing their children’s exposure to pesticides, despite being aware of the dangers. This phenomenon illustrates what is known as the “Intention–Behavior Gap” in social psychology literature [44]. This finding contrasts with the results of a study by Khan et al. [45] in Pakistan, which found that most farmers felt fully responsible for the safety of their children from pesticide exposure. This difference indicates contextual variation in the internalization of personal norms, which may be influenced by cultural factors, education level, and access to health information [43].

Although awareness of health risks is increasing, other factors such as habits, limited knowledge, and lack of adequate system support often hinder the implementation of stronger preventive measures [46,47]. Furthermore, 48.8% of the respondents regretted having their children experience health problems due to pesticide exposure. Although farming families experience the emotional impact of pesticide use, protecting children remains a low priority. In other words, despite practical responses often being more influenced by routines and familiar practices [48].

### 4.2. Long-Term and Short-Term Goals

This study also found that long-term goals did not significantly influence pesticide use patterns (β = −0.073; *p* = 0.256), in contrast to the findings of Kaiser et al. [17], who found a significant negative relationship between long-term goals and pesticide use (*p* < 0.05). This phenomenon reflects the tension between short-term economic goals and long-term conservation objectives, as described in previous research. A total of 35.8% of respondents stated they wanted to do so “always,” and 27.4% stated they wanted to do so “often,” indicating a strong desire to make changes toward more sustainable agriculture. Daugbjerg [49] also confirmed that aspirations toward pesticide-free farming are growing. These findings strengthen the argument that well-targeted educational interventions can play a catalytic role in accelerating shifts in mindset and practice among farmers by helping them see that child protection and economic goals do not have to be incompatible.

The desire to pass on productive and safe land to children also indicates a deep concern for future generations. Although only 41.4% of respondents “always” and 20.8% “often” felt this way, this data demonstrates a significant desire to protect the environment and ensure the long-term sustainability of family livelihoods. Sukayat et al. [50] emphasized that despite the desire to pass on safer and more productive land, farmers often face practical constraints that influence their decisions regarding chemical use and other farming methods. These constraints can include immediate economic pressures, limited access to alternative inputs, and a lack of technical knowledge about child-safe farming methods.

Short-term goals, as the only individual-level variable, negatively influenced pesticide use patterns (β = −0.178; *p* = 0.018). This finding aligns with Kaiser et al. [17], who found that pesticide use patterns were significantly influenced by short-term goals (*p* < 0.05). This negative effect indicates that the greater the farmers’ concern for protecting children’s health in the short term, the lower the intensity or the safer the pesticide use practices they implemented. This study found that respondents tended to prioritize more direct and immediate risks, such as those related to consumption, 61.4% always wanted to ensure food given to children was free of pesticide residues, over more indirect risks, such as dermal contact in play areas. A study by Shekhar et al. [51] showed that in the context of health risks, people are more likely to respond to threats with immediate impacts, such as those from food consumption or pesticide inhalation, compared to indirect risks, such as dermal exposure during play. People tend to be more consistent in their desire to protect children from exposure to direct health risks, as these are easier to understand and control in everyday contexts. From a behavior change perspective, this finding is important because when the issue is framed in an immediate, tangible, and actionable context, respondents demonstrate a much stronger and more consistent intention to protect.

### 4.3. Social Norms

Social norms within farming communities did not influence pesticide use patterns in this study. This finding is inconsistent with that of Ndirangu et al. [52], who emphasized the positive influence of social norms, particularly cultural and peer factors, on safe pesticide use behavior. This is consistent with previous research, which also found that social norms are negatively related to pesticide use patterns [17]. The data show that awareness of the risks of safe pesticide use still varies among respondents. The majority of respondents (27.9%) stated that they sometimes wish they had not used dangerous pesticides, were never advised by their colleagues to be careful when using pesticides (26.5%), and were never advised not to spray near children. These findings indicate that social norms at the research site do not optimally support safe pesticide use practices for children. This condition aligns with research by Satya Sai [53], who found that although awareness of the dangers of pesticides is increasing, social norms and inherited habits in farming communities still hamper safe use practices. Social norms should also motivate farmers to internalize the use of safer practices to avoid reputational loss or social isolation. This can occur when they observe their neighbors and friends using such methods or when they realize that their colleagues expect them to adopt them [54]. Social norms can be a key motivator for them to adopt safer agricultural practices [55], not only for the environment but also for vulnerable groups, such as children.

### 4.4. Competencies

Complementing the role of social norms, farmers’ technical competence—particularly their knowledge and application of alternative pest management strategies—represents another structural determinant of pesticide use behavior. The practice of using organic pesticides, particularly the cultivation of pest-repellent plants, is the competency aspect that significantly influences pesticide use patterns. The results of this study support the research of Gesesew et al. [56] that integrated pest management practices are one predictor of safe pesticide use. Chemical pesticides are often the primary choice for farmers due to their convenience and proven effectiveness, despite the potential for adopting more environmentally friendly alternatives [19].

Many farmers still rely on chemical pesticides because their farming systems have become accustomed to their use, and the time and support required to transition to more environmentally friendly alternatives. This study also shows that barriers to switching to alternative methods lie not only in economic factors but also in the need to broaden knowledge and mindsets about more proactive pest management. Pududu & Rother [57] suggested that to introduce alternative methods more widely, providing adequate training and resources and building confidence that these methods can produce good results are crucial. Education that integrates a family health perspective, particularly protecting children from toxic exposures, can be a powerful motivator for farmers to adopt safer practices. Strategies to mitigate the perceived risk of pesticide exposure in children should be an integral component of agricultural extension programs, emphasizing that protecting children’s health is not only a public health responsibility but also part of sustainable agricultural practices that protect future generations.

### 4.5. Materials (Children’s Healthcare Costs)

In addition to competence, the material or economic dimension, specifically the perceived financial burden of healthcare costs for children, constitutes a significant motivational factor in shaping pesticide use decisions. The materiality represented by children’s medical costs was shown to have a positive effect on safer pesticide use patterns (β = 0.263; *p* = 0.003), consistent with Rufo et al. [58] but contradicting Ndirangu [52]. This difference suggests that concern about children’s health costs can motivate farmers’ behavioral changes, although financial constraints can generally reduce compliance with pesticide safety practices. The striking disparity between high pesticide costs and low child health costs suggests a potential misperception among farmers. This perception, as found by several previous studies, is that the health costs of pesticide use are negligible compared to the increased productivity gained from pesticide use [58]. However, this economic interpretation potentially overlooks several important dimensions of actual health costs. Low recorded medical costs may not reflect the true health burden but rather reflect underutilization of health services or a lack of recognition of health symptoms associated with pesticide exposure. The high cost of pesticides also indicates potential household economic vulnerability [58]. When nearly a quarter of the crop value is allocated to pesticide purchases, farming families become vulnerable to fluctuations in agricultural input and output prices. These findings underscore the need for more comprehensive cost–benefit analyses that consider not only short-term costs and benefits but also integrate long-term projected health costs and environmental impacts.

### 4.6. Pesticide Use Patterns

Taken together, the cumulative effect of these determinants—spanning individual, social, technical, and economic dimensions—ultimately manifests in pesticide use patterns that have direct consequences for children’s health. This study found that pesticide use patterns influence the pesticide exposure risk in children, with the primary indicator being the insecticide dosage. These findings support the findings of a previous study that found that the pesticide exposure risk is influenced by pesticide use patterns, including dosage, duration, and frequency [5]. Mismatches in pesticide dosages with recommended dosages were found in the practices of several respondents. These findings are consistent with several previous studies that reported inappropriate pesticide application practices among farmers. Yuantari et al. [59] found that 40.7% of farmers used more than 10 types of active ingredients in a single mixture, and 51.9% of farmers sprayed 6–10 tanks per day. Previous studies have indicated that this mismatch in dosage was closely related to farmers’ lack of knowledge about how to calculate the correct concentration [60]. The difference with this study lies in the larger range of land areas (up to 10.50 hectares), which indicates the existence of a segment of farmers with a larger business scale but still implementing similar practices. The implications of this finding are serious, considering that pesticide overdose can cause residue accumulation in the soil that lasts for years [61], while chronic exposure can increase the risk of neurological disorders in children living in agricultural areas [62,63,64]. Therefore, an approach based on clear, immediately applicable actions is more likely to produce tangible changes in the protection of children from pesticide hazards. Overall, these findings suggest that an approach based on practical, immediate, short-term actions will be more effective in driving community behavior change regarding pesticide use, particularly in protecting children from more immediate risks.

### 4.7. Limitations

From a health perspective, toddler health data should ideally be obtained from clinical medical records or specific screening results for toxic exposure. However, these clinical data were inaccessible due to the absence of a routine toddler poisoning screening program in the study area, as well as limited access to medical records at local health facilities with underdeveloped digital record systems. Most farming families also rarely take toddlers to health facilities for minor complaints, so not all toddler health conditions are recorded in the formal health care system. Therefore, this study utilized data on toddler illness reported by parents, while acknowledging that such data do not provide definitive evidence that the symptoms were caused by a specific toxic exposure. Symptoms such as fever, cough, vomiting, and respiratory distress can be caused by various other factors, including infections, environmental conditions, dietary factors, or endemic diseases in the area. However, considering the parents’ work history as potato farmers with high pesticide use, the proximity of the households to the fields, and the toddler’s frequency of visits to agricultural areas, the risk of pesticide exposure remains an important variable that cannot be ignored in the analysis of toddler health outcomes. Furthermore, the low R^2^ value of 2.9% for the childhood pesticide exposure risk outcome suggests that the variables included in this model explain only a small portion of the variance in toddler health symptoms, which is consistent with the complexity of child health determinants. These findings should be interpreted with caution, as toddler health outcomes are likely influenced by many unmeasured factors beyond pesticide exposure. Future studies are encouraged to include clinical biomarkers and a broader set of health determinants to increase the model’s explanatory power.

## 5. Conclusions

This study found that pesticide use patterns influenced the pesticide exposure risk in children, with insecticide dose as the main indicator. From the socio-ecological aspect, competence in the form of organic pesticide use practices showed the strongest influence on pesticide use patterns, followed by materials in the form of child medical costs and short-term goals. From the individual aspect, personal values were the most dominant predictors of personal norms and long-term goals. Interestingly, social norms only influenced the formation of personal norms but not directly on the patterns of pesticide use. Similarly, self-efficacy, personal norms, and long-term goals did not significantly influence pesticide use patterns. To address the dominant influence of competence, intervention programs should prioritize technical training on calculating appropriate pesticide dosages and integrated pest management. Health communication approaches should be framed in the context of concrete, immediate, short-term protective actions—such as ensuring that food is free of pesticide residues—as they have been shown to be more effective than abstract, long-term advocacy for change. Regulations and training for pesticide distributors are needed to convey safety information based on children’s health. Agricultural extension services must integrate a family health perspective. Communication strategies should leverage farmers’ personal values, particularly the desire to protect children, as leverage to encourage behavior change while building new social norms within farming communities that support safe practices through peer education and demonstration plots. Spatial interventions such as buffer zones and the separation of pesticide application areas from children’s activity areas are also needed.

## Figures and Tables

**Table 1:** Research Variables and Indicators.

Variable Code	Variables	Indicator Code	Indicator
X1	Self-Efficacy	X11	Beliefs about preventing children’s exposure to pesticides
		X12	Belief in rejecting the use of dangerous pesticides
		X13	Confidence in knowing the safe time for spraying
		X14	Confidence in storing pesticides safely
X2	Personal Values	X21	Concern for child safety
		X22	Discomfort due to pesticide exposure in children
		X23	Commitment to farming in a safe way
		X24	Hope for children free from the dangers of pesticides
X3	Competence	C11	Knowledge of the impact of pesticides on child development
		C12	Knowledge of safe pesticide storage
		C13	Knowledge of exposure routes through the skin
		C14	Knowledge of cancer risks due to pesticides
		C15	Knowledge of behavioral impacts due to pesticides
		C16	Knowledge of actions in case of poisoning
		C21	Practice of using natural pest repellent ingredients
		C22	Practice of planting pest repellent plants
		C23	Dependence on chemical pesticides
		C24	Routine checks of plant conditions
		C25	Clean the garden from plant residue
		C26	Clearing the land of weeds before planting
X4	Social Norms	SN11	Family expectations regarding safe pesticide use
		SN12	Peer support in the careful use of pesticides
		SN13	Neighbors advise not to spray near children
		SN14	Public views on safe pesticide storage
		SN21	Farmers’ habits in using pesticides
		SN22	Farmers’ habits in storing pesticides
		SN23	Habits of using personal protective equipment (PPE)
		SN24	The habit of spraying near children
X5	Short Term Goals	X51	Protecting children’s health from pesticides
		X52	Children’s food safety from pesticide residues
		X53	Safety of children’s play areas from pesticides
		X54	Reducing children’s direct contact with pesticides
X6	Material	M1	Yield value per planting season
		M2	Expenditure for purchasing pesticides
		M3	Child medical expenses due to health problems
Y1	Personal Norms	Y11	Guilt about using dangerous pesticides
		Y12	Discomfort in spraying near children
		Y13	Responsibility for preventing pesticide exposure in children
		Y14	Regret over the impact of pesticides on children
		Y15	Awareness of children’s health risks due to pesticides
Y2	Long Term Goals	Y21	Hope for a pesticide-free agricultural environment
		Y22	Commitment to environmentally friendly agricultural systems
		Y23	The desire to inherit safe and productive land
		Y24	A sustainable view of family farming practices
Y3	Pesticide Use Patterns	PPP21	Insecticide dosage per hectare
		PPP22	Dosage of herbicide use per hectare
		PPP23	Dosage of fungicide use per hectare
		PPP31	Frequency of insecticide use per planting season
		PPP32	Frequency of herbicide use per growing season
		PPP33	Frequency of fungicide use per growing season
Y4	Pesticide Exposure Risk in Children	PRT1	Exposure of children in agricultural areas during spraying
		PRT21	Permission for children to enter the land after spraying
		PRT22	Prohibition of playing in the spray equipment washing area
		PRT23	The habit of bathing and changing clothes after spraying
		PRT24	Marking of risk areas after spraying
		PRT25	Separation of work clothes washing from children’s clothes
		PRT3	Self-Reported Symptoms Associated with Pesticide Exposure (cough, fever, nausea, skin irritation)

Note: Variable Code (e.g., X1) denotes the latent construct, while Indicator Code (e.g., X11–X14) denotes its observed measurement indicators.

**Table 2:** Respondent characteristics.

Variables	Sub Variables	Amount (n = 215)	Percentage (n = 100)
Number of children	1	194	90.2
	>1	21	9.8
Length of work	≤10 Years	96	44.7
	>10 Years	119	55.3
Last education	Never Went to School	10	4.7
	Did Not Graduate from Elementary School	24	11.2
	Elementary school	91	42.3
	Junior high school	45	20.9
	High School/Senior High School	38	17.7
	Diploma	1	0.5
	College	6	2.8
Main Job	Farmer	206	95.8
	Non-Farmer	9	4.2
Source of Knowledge	Friend	83	38.6
	Agricultural Extension Worker	52	24.2
	Pesticide Seller	98	45.6
	Family	56	26.0
	Social media	3	1.4
Age	<20 Years	9	4.2
	20–24 Years	18	8.4
	25–29 Years	135	62.8
	30–34 Years	20	9.3
	35–39 Years	11	5.1
	≥40 Years	27	12.6
Distance from Home	0–100 m	194	90.2
	101–500 m	13	6.2
	501–1000 m	8	3.7
Location of Toddlers During Pesticide Spraying	At home	178	83.0
	In the field	11	5.0
	In field hut/farm house	26	12.0
Pesticide Storage Place	Hung inside the house	19	9.0
	Placed in the corner of the house	19	9.0
	In field hut/farm house	92	43.0
	In warehouse	80	37.0
	Outside the house	5	2.0

**Table 3:** Self-Reported Health Symptoms in Respondents’ Children.

Disease History	Yes		No	
	n	%	n	%
Acute Respiratory Infection (ARI)	3	1.4%	212	98.6%
Vomiting	53	24.7%	162	75.3%
Nausea	18	8.4%	197	91.6%
Cough	125	58.1%	90	41.9%
Fever	135	62.8%	80	37.2%
Dermatitis	14	6.5%	201	93.5%
Asthma	5	2.3%	210	97.7%
Seizure	8	3.7%	207	96.3%
Speech delay	2	0.9%	213	99.1%

**Table 4:** Results of validity and reliability tests of the initial model.

	Initial Model		Final Model	
	Composite Reliability (rho_c)	Average Variance Extracted (AVE)	Composite Reliability (rho_c)	Average Variance Extracted (AVE)
X1	0.756	0.469	0.822	0.606
X2	0.837	0.565	0.852	0.657
X3	0.620	0.176	0.808	0.679
X4	0.506	0.193	0.843	0.729
X5	0.891	0.672	0.885	0.720
X6	0.764	0.615	0.959	0.922
Y1	0.750	0.389	0.845	0.731
Y2	0.683	0.364	0.823	0.703
Y3	0.531	0.241	0.759	0.616
Y4	0.567	0.186	0.808	0.679

**Table 5:** Final model R-square results.

	R-Square	R-Square Adjusted
Y1	0.473	0.466
Y2	0.214	0.211
Y3	0.172	0.148
Y4	0.029	0.025

**Table 6:** Results of the final model path coefficient test (bootstrapping).

	Original Sample (O)	Sample Mean (M)	Standard Deviation (STDEV)	T Statistics (|O/STDEV|)	*p* Values	Conclusion
X1 → Y1	0.121	0.127	0.070	1.724	0.085	H1 rejected—Not Significant
X2 → Y1	0.395	0.392	0.082	4.835	0.000	H2 (a) is accepted—Significant
X2 → Y2	0.463	0.471	0.056	8.275	0.000	H2 (b) is accepted—Significant
Y2 → Y3	−0.073	−0.073	0.064	1.136	0.256	H3 rejected—Not Significant
X5 → Y3	−0.178	−0.189	0.076	2.357	0.018	H4 accepted—Significant
Y1 → Y3	0.090	0.092	0.099	0.905	0.365	H5 rejected—Not Significant
X4 → Y3	0.050	0.056	0.074	0.672	0.501	H6 (a) is rejected—Not Significant
X4 → Y1	0.290	0.291	0.059	4.893	0.000	H6 (b) is accepted—Significant
X3 → Y3	−0.553	−0.590	0.168	3.280	0.001	H7 accepted—Significant
X6 → Y3	0.263	0.269	0.088	2.987	0.003	H8 accepted—Significant
Y3 → Y4	−0.171	−0.188	0.058	2.952	0.003	H9 accepted—Significant

## Data Availability

The original data presented in the study are openly available at: https://drive.google.com/uc?export=download&id=1fYRW83zky3tzwrGbADdgighAdn2dW-rV (accessed on 10 June 2026).

## Supplementary Materials

The following supporting information can be downloaded at: https://www.mdpi.com/article/10.3390/jox16030117/s1, Table S1: Results of the Discriminant Validity Test of Latent Variables; Table S2: Results of the Discriminant Validity Test of Indicators (Cross Loadings); Table S3: Final Outer Model Results—VIF Values; Table S4: Final Model Fit Results.

## Abbreviations

The following abbreviations are used in this manuscript:

AVE	Average Variance Extracted
NFI	Normal Fit Index
SEM-PLS	Structural Equation Modeling-Partial Least Squares
SRMR	Standardized Root Mean Square Residual

## References

[1] Unsworth J. History of Pesticide Use. http://agrochemicals.iupac.org/index.php?option=com_sobi2&sobi2Task=sobi2Details&catid=3&sobi2Id=31.

[2] Bernardes M.F.F., Pazin M., Pereira L.C., Dorta D.J., Andreazza A.C., Scola G. (2015). Impact of Pesticides on Environmental and Human Health.

[3] Benbrook C., Perry M.J., Belpoggi F., Landrigan P.J., Perro M., Mandrioli D., Antoniou M.N., Winchester P., Mesnage R. (2021). Commentary: Novel strategies and new tools to curtail the health effects of pesticides. Environ. Health.

[4] Furlong C.E., Holland N., Richter R.J., Bradman A., Ho A., Eskenazi B. (2006). PON1 status of farmworker mothers and children as a predictor of organophosphate sensitivity. Pharmacogenet. Genom..

[5] Davies B., Hlela M.B.K.M., Rother H.A. (2023). Child and adolescent mortality associated with pesticide toxicity in Cape Town, South Africa, 2010–2019: A retrospective case review. BMC Public Health.

[6] Ibrahim I., Sillehu S. (2022). Risk Behavior for Pesticide Exposure in Children living in Agricultural Area. Divers. Dis. Prev. Res. Integr..

[7] Robests J.R., Karr C.J., Council on Environmental Health (2012). Pesticide Exposure in Children. Pediatrics.

[8] Kachaiyaphum P., Howteerakul N., Sujirarat D., Siri S., Suwannapong N. (2010). Serum cholinesterase levels of Thai chilli-farm workers exposed to chemical pesticides: Prevalence estimates and associated factors. J. Occup. Health.

[9] Kaushal J., Khatri M., Arya S.K. (2021). A treatise on Organophosphate pesticide pollution: Current strategies and advancements in their environmental degradation and elimination. Ecotoxicol. Environ. Saf..

[10] Tutu C.G., Manampiring A.E., Umboh A. (2020). Faktor-Faktor yang Berhubungan dengan Aktivitas Enzim Cholinesterase Darah pada Petani Penyemprot Pestisida. J. Public Health Community Med..

[11] Ye M., Beach J., Martin J.W., Senthilselvan A. (2013). Occupational pesticide exposures and respiratory health. Int. J. Environ. Res. Public Health.

[12] Park A.S., Ritz B., Yu F., Cockburn M., Heck J.E. (2020). Prenatal pesticide exposure and childhood leukemia—A California statewide case-control study. Int. J. Hyg. Environ. Health.

[13] Patel D.M., Gyldenkærne S., Jones R.R., Olsen S.F., Tikellis G., Granström C., Dwyer T., Stayner L.T., Ward M.H. (2020). Residential proximity to agriculture and risk of childhood leukemia and central nervous system tumors in the Danish national birth cohort. Environ. Int..

[14] Bourguet D., Guillemaud T., Lichtfouse E. (2016). The Hidden and External Costs of Pesticide Use BT—Sustainable Agriculture Reviews: Volume 19.

[15] Marquez E.C., Schafer K.S. (2016). Kids Frontline—How Pesticides Are Undermining the Health of Rural Children.

[16] Shove E., Sage (2012). The Dynamics of Social Practice.

[17] Kaiser A., Robin S., Burger P. (2024). Toward a low-pesticide agriculture: Bridging practice theory and social-psychological concepts to analyze farmers’ routines. Sustain. Sci. Pract. Policy.

[18] Schmidt P., Tatarko A. (2016). Entrepreneurial Intention and Values: Results from a Russian Population Survey. Psychol. J. High. Sch. Econ..

[19] Tudi M., Ruan H.D., Wang L., Lyu J., Sadler R., Connell D., Chu C., Phung D.T. (2021). Agriculture development, pesticide application and its impact on the environment. Int. J. Environ. Res. Public Health.

[20] Bandura A. (1997). Behavior theory and the models of man (1974). The Evolution of Psychology: Fifty Years of the American Psychologist.

[21] Sapkota U., Bhandari G., Sapkota M., Khanal S., Poudel A., Khanal D., Pokhrel M.R., Damalas C.A. (2025). Modeling vegetable farmers’ intention to use pesticides in central Nepal: An extended version of the planned behavior theory. Environ. Chall..

[22] Yang Q., Al Mamun A., Reza M.N.H., Naznen F., Masud M.M. (2024). Modeling the intention and usage of organic pesticide control using value-belief-norm model. Environ. Res. Commun..

[23] Klekotko M., Klekotko M. (2024). Urban Scenes as Community Practices BT—Scenes and Communities in the City.

[24] Yang X., Wang F., Meng L., Zhang W., Fan L., Geissen V., Ritsema C.J. (2014). Farmer and retailer knowledge and awareness of the risks from pesticide use: A case study in the Wei River catchment, China. Sci. Total Environ..

[25] Busco C. (2009). Giddens’ structuration theory and its implications for management accounting research. J. Manag. Gov..

[26] Zhang J., Li B., Zhang Y., Gong C., Liu Z. (2022). From Entrepreneurship Education, Government Support, and Global Competence to Entrepreneurial Behavior: The Serial Double Mediating Effect of the Self-Efficacy and Entrepreneurial Intention. Front. Psychol..

[27] Quirós-Alcalá L., Bradman A., Nishioka M., Harnly M.E., Hubbard A., McKone T.E., Ferber J., Eskenazi B. (2011). Pesticides in house dust from urban and farmworker households in California: An observational measurement study. Environ. Health.

[28] FAO (2017). The Future of Food and Agriculture: Trends and Challenges.

[29] Van Niejenhuis J.H., Wossink G.A.A., Struik P.C., Vredenberg W.J., Renkema J.A., Parlevliet J.E. (1994). Review and Application of the Farming Styles Concept: The Case of Dutch Arable Farming BT—Plant Production on the Threshold of a New Century: Proceedings of the International Conference at the Occasion of the 75th Anniversary of the Wageningen Agricult.

[30] Meunier E., Smith P., Griessinger T., Robert C. (2024). Understanding changes in reducing pesticide use by farmers: Contribution of the behavioural sciences. Agric. Syst..

[31] BPS-Statistics Gowa Regency (2024). Kecamatan Tinggimoncong Dalam Angka.

[32] BPS Produksi Kentang Provinsi Sulawesi Selatan Menurut Kabupate/Kota (Kuintal), 2018–2020. https://sulsel.bps.go.id/indicator/55/1100/1/kentang-.html.

[33] Arifin A.M. (2019). Efisiensi Teknis Usahatani Kentang di Kabupaten Gowa Sulawesi Selatan. Forum Agribisnis.

[34] Xie S., Hofmann J.N., Sampson J.N., Josse P.R., Madrigal J.M., Chang V.C., Deziel N.C., Andreotti G., Keil A.P., Ward M.H. (2024). Quantitative measures of recent and lifetime agricultural pesticide use are associated with increased pesticide concentrations in house dust. Environ. Int..

[35] Lu C., Fenske R.A., Simcox N.J., Kalman D. (2000). Pesticide exposure of children in an agricultural community: Evidence of household proximity to farmland and take home exposure pathways. Environ. Res..

[36] Tessier N., Boissonnot R., Desvignes V., Fröchen M., Merlo M., Blanchard O., Chevrier C., Guldner L., Mandin C., Yamada O. (2023). Use and storage of pesticides at home in France (the Pesti’home survey 2014). Environ. Res..

[37] Al-Dawood A., Shawaqfeh S., Al-Zyoud F., Mamkagh A., Al-Atiyat R., Hasan H. (2023). Awareness of pesticides’ residues in food and feed among students of the Faculty of Agriculture, Mutah University, Jordan. J. Saudi Soc. Agric. Sci..

[38] Swartz A., Levine S., Rother H.A., Langerman F. (2018). Toxic layering through three disciplinary lenses: Childhood poisoning and street pesticide use in Cape Town, South Africa. Med. Humanit..

[39] Deng Y., Dai H., Zeng M., Guan L., Luo X., Zhang C., Tian J., Zhang J., Li Y., Xi Q. (2019). Knowledge and behavior regarding pesticide use: A survey among caregivers of children aged 1–6 years from rural China. Environ. Sci. Pollut. Res..

[40] Hair J., Alamer A. (2022). Partial Least Squares Structural Equation Modeling (PLS-SEM) in second language and education research: Guidelines using an applied example. Res. Methods Appl. Linguist..

[41] Hair J., Hollingsworth C.L., Randolph A.B., Chong A.Y.L. (2017). An updated and expanded assessment of PLS-SEM in information systems research. Ind. Manag. Data Syst..

[42] Rezaei R., Safa L., Damalas C.A., Ganjkhanloo M.M. (2019). Drivers of farmers’ intention to use integrated pest management: Integrating theory of planned behavior and norm activation model. J. Environ. Manag..

[43] Eskandari A., Fatemi M. (2025). Pro-environmental behavior in agriculture: Exploring VBN-theory-based drivers among summer crop farmers in Southern Iran. Environ. Sustain. Indic..

[44] Sheeran P., Webb T.L. (2016). The Intention–Behavior Gap. Soc. Personal. Psychol. Compass.

[45] Khan M.I., Shoukat M., Alam S., Arif H., Niazi N., Azam M., Bashir S., Ashraf I., Qadri R. (2020). Use, Contamination and Exposure of Pesticides in Pakistan: A Review. Pak. J. Agric. Res..

[46] Chekol G.M. (2025). Understanding the factors behind non-adherence to pesticide safety guidelines among smallholder farmers in Fogera and MEcha districts, northwestern Ethiopia. BMC Res. Notes.

[47] Salmani S., Mousavi S.H., Navardi S., Hosseinzadeh F., Pashaeypoor S. (2022). The barriers and facilitators to health-promoting lifestyle behaviors among people with multiple sclerosis during the coronavirus disease 2019 pandemic: A content analysis study. BMC Neurol..

[48] Albarracín D., Fayaz-Farkhad B., Samayoa J.A.G. (2024). Determinants of behaviour and their efficacy as targets of behavioural change interventions. Nat. Rev. Psychol..

[49] Daugbjerg C. (2023). Using public procurement of organic food to promote pesticide-free farming: A comparison of governance modes in Denmark and Sweden. Environ. Sci. Policy.

[50] Sukayat Y., Setiawan I., Suharfaputra U., Kurnia G. (2023). Determining Factors for Farmers to Engage in Sustainable Agricultural Practices: A Case from Indonesia. Sustainability.

[51] Shekhar C., Khosya R., Thakur K., Mahajan D., Kumar R., Kumar S., Sharma A.K. (2024). A systematic review of pesticide exposure, associated risks, and long-term human health impacts. Toxicol. Rep..

[52] Ndirangu Z.W., Zoltan E., Muhoro A.M. (2025). Determinants of Pesticide Safety Adherence among Tomato Farmers in Kenya: Individual and Structural Factors for Sustainable Agriculture. J. Sustain. Res..

[53] Satya Sai M.V., Revati G.D., Ramya R., Swaroop A.M., Maheswari E., Kumar M.M. (2019). Knowledge and Perception of Farmers Regarding Pesticide Usage in a Rural Farming Village, Southern India. Indian J. Occup. Environ. Med..

[54] Li X., Wu X. (2021). The impact of social norms on rice farmers’ behavior of organic fertilizers application: Mediating effect of value perception and moderating effect of education level. Int. J. Low-Carbon Technol..

[55] Ambrosius F.H.W., Kramer M.R., Spiegel A., Bokkers E.A.M., Bock B.B., Hofstede G.J. (2022). Diffusion of organic farming among Dutch pig farmers: An agent-based model. Agric. Syst..

[56] Gesesew H.A., Woldemichael K., Massa D., Mwanri L. (2016). Farmers knowledge, attitudes, practices and health problems associated with pesticide use in rural irrigation villages, Southwest Ethiopia. PLoS ONE.

[57] Pududu B.A., Rother H.A. (2021). Whose jurisdiction is home contamination? Para-occupational ‘take-home’ herbicide residue exposure risks among forestry workers’ families in south africa. Int. J. Environ. Res. Public Health.

[58] Rufo E., Brouwer R., van Beukering P. (2024). The social costs of pesticides: A meta-analysis of the experimental and stated preference literature. Sci. Rep..

[59] Yunarti M.G.C., Widianarko B., Sunoko R.H. (2015). Analisis risiko pajanan pestisida terhadap kesehatan petani. J. Kesehat. Masy..

[60] Istriningsih, Dewi Y.A., Yulianti A., Hanifah V.W., Jamal E., Dadang, Sarwani M., Mardiharini M., Anugrah I.S., Darwis V. (2022). Farmers’ knowledge and practice regarding good agricultural practices (GAP) on safe pesticide usage in Indonesia. Heliyon.

[61] Alaoui A., Christ F., Abrantes N., Silva V., González N., Gai L., Harkes P., Navarro I., de la Torre A., Martínez M.Á. (2024). Assessing pesticide residue occurrence and risks in the environment across Europe and Argentina. Environ. Pollut..

[62] Wołejko E., Łozowicka B., Jabłońska-Trypuć A., Pietruszyńska M., Wydro U. (2022). Chlorpyrifos Occurrence and Toxicological Risk Assessment: A Review. Int. J. Environ. Res. Public Health.

[63] Khan K.M., Gaine M.E., Daniel A.R., Chilamkuri P., Rohlman D.S. (2024). Organophosphorus pesticide exposure from house dust and parent-reported child behavior in Latino children from an orchard community. Neurotoxicology.

[64] Rodríguez A., Castrejón-Godínez M.L., Monterrosas-Brisson N. (2025). Pesticides: Environmental Stressors Implicated in the Development of Central Nervous System Disorders and Neurodegeneration. Stresses.

