# Supplementary material for: Preventing Pesticide Toxicity Risk Through Self-Reported Practices in Children of Farming Communities: A Social Practice Theory Perspective
Source: J Xenobiot. 2026 Jun 22;16(3):117. doi: 10.3390/jox16030117 (PMC13302243; doi:10.3390/jox16030117)
Supplement: Supplementary file 1 [file jox-16-00117-s001.zip › jox-4337610-supplementary.pdf]

Supplementary Materials: Preventing Pesticide Toxicity Risk Through Self-Reported Practices in Children of Farming Communities: A Social Practice Theory Perspective

Nuraeni Nuraeni, Herdis Herdiansyah, Fatmah Fatmah, Haruki Agustina and Rully Yusuf

Table S1. Results of *the Discriminant Validity Test* of Latent Variables

	X1	X2	X3	X4	X5	X6	Y1	Y2	Y3	Y4
X1	**0.778**									
X2	0.609	**0.751**								
X3	0.116	0.069	**0.737**							
X4	0.477	0.551	-0.049	**0.854**						
X5	0.541	0.689	0.067	0.486	**0.858**					
X6	-0.117	-0.139	-0.210	-0.024	-0.159	**0.945**				
Y1	0.500	0.629	-0.046	0.566	0.537	-0.013	**0.855**			
Y2	0.283	0.463	-0.030	0.277	0.419	-0.125	0.386	**0.839**		
Y3	-0.069	-0.143	-0.242	-0.004	-0.190	0.335	-0.002	-0.127	**0.811**	
Y4	0.083	0.107	0.299	0.001	0.056	-0.090	-0.024	-0.041	-0.171	**0.773**

Table S2. Results of the Discriminant Validity Test of Indicators (Cross Loadings)

	X1	X2	X3	X4	X5	X6	Y1	Y2	Y3	Y4
**C21**	-0.041	-0.011	**0.756**	-0.067	-0.002	-0.127	-0.046	-0.012	-0.175	0.180
**C22**	0.181	0.057	**0.882**	-0.056	0.029	-0.066	-0.046	-0.043	-0.222	-0.035
**M1**	-0.033	-0.058	-0.113	0.051	-0.026	**0.964**	-0.001	-0.047	0.386	-0.100
**M3**	-0.095	-0.117	-0.098	-0.022	-0.115	**0.956**	-0.097	-0.131	0.351	-0.147
**PPP1**	-0.104	-0.241	-0.202	0.006	-0.175	0.315	-0.061	-0.162	**0.832**	-0.107
**PPP21**	0.009	0.041	-0.195	-0.017	-0.064	0.311	0.084	-0.027	**0.794**	-0.036
**PRT23**	0.141	0.113	0.047	0.236	0.088	-0.127	0.255	0.141	-0.094	**0.981**
**PRT25**	0.098	0.110	0.114	0.135	0.087	-0.060	0.175	0.037	-0.022	**0.543**
**SN14**	0.319	0.491	-0.129	**0.847**	0.498	0.003	0.473	0.305	0.003	0.166
**SN22**	0.492	0.404	0.002	**0.861**	0.342	0.026	0.493	0.166	-0.013	0.244
**X11**	**0.810**	0.519	-0.031	0.448	0.522	0.036	0.467	0.203	0.014	0.035
**X13**	**0.755**	0.384	0.172	0.250	0.384	-0.093	0.319	0.256	-0.146	0.182
**X14**	**0.769**	0.423	0.154	0.384	0.341	-0.127	0.355	0.207	-0.041	0.161
**X22**	0.614	**0.795**	0.038	0.534	0.561	-0.025	0.579	0.371	-0.089	0.208
**X23**	0.388	**0.855**	0.031	0.384	0.511	-0.111	0.500	0.466	-0.145	0.062
**X24**	0.386	**0.781**	0.010	0.337	0.474	-0.085	0.391	0.355	-0.079	0.015
**X52**	0.496	0.578	-0.046	0.382	**0.823**	-0.058	0.457	0.433	-0.081	0.046
**X53**	0.422	0.515	0.035	0.414	**0.923**	-0.039	0.433	0.399	-0.170	0.096
**X54**	0.530	0.584	0.036	0.465	**0.777**	-0.102	0.502	0.422	-0.097	0.094
**Y11**	0.353	0.495	-0.089	0.477	0.386	0.072	**0.838**	0.258	0.065	0.234
**Y15**	0.495	0.552	-0.010	0.491	0.520	-0.143	**0.873**	0.390	-0.042	0.224
**Y22**	0.090	0.260	-0.054	0.077	0.253	-0.107	0.149	**0.742**	-0.154	0.078
**Y23**	0.325	0.517	-0.018	0.325	0.505	-0.062	0.430	**0.928**	-0.075	0.138

Table S3. Final Outer Model Results - VIF Values

Indicator	VIF
C21	1.188
C22	1.232
C23	1.168
M1	2.643
M3	2.643
PPP21	1.117
PPP23	1.117
PRT1	1.044
PRT21	1.044
SN14	1.266
SN22	1.266
X11	1.240
X13	1.405
X14	1.391
X21	1.367
X22	1.666
X23	1.692
X24	1.562
X51	1.748
X52	2.039
X53	1.982
Y11	1.276
Y15	1.276
Y22	1.239
Y23	1.239

Table S4. Final Model Fit Results

	Saturated model	*Estimated model*
SRMR	0.086	0.098
d_ULS	2.388	3.142
d_G	0.811	1.050
Chi-square	1083.849	1571.398
NFI	0.486	0.255
